# Abortion trends from 1996 to 2011 in Estonia: special emphasis on repeat abortion

**DOI:** 10.1186/1472-6874-14-81

**Published:** 2014-07-09

**Authors:** Made Laanpere, Inge Ringmets, Kai Part, Kärt Allvee, Piret Veerus, Helle Karro

**Affiliations:** 1 Department of Obstetrics and Gynaecology, University of Tartu, L. Puusepa 8, Tartu 51014, Estonia; 2 Department of Public Health, University of Tartu, Ravila 19, Tartu 50411, Estonia; 3 Tartu University Hospital Women's Clinic, L. Puusepa 8, Tartu 51014, Estonia; 4 Estonian Medical Birth and Abortion Registry. National Institute for Health Development, Hiiu 42, Tallinn 11619, Estonia; 5 Department of Epidemiology and Biostatistics. National Institute for Heath Development, Hiiu 42, Tallinn 11619, Estonia

**Keywords:** Induced abortion, Repeat abortion, Abortion trends, Estonian Abortion Registry, Contraception

## Abstract

**Background:**

The study aimed to describe the overall and age-specific trends of induced abortions from 1996 to 2011 with an emphasis on socio-demographic characteristics and contraceptive use of women having had repeat abortions in Estonia.

**Methods:**

Data were retrieved from the Estonian Medical Birth and Abortion Registry and Statistics Estonia. Total induced abortion numbers, rates, ratios and age-specific rates are presented for 1996–2011. The percentage change in the number of repeat abortions within selected socio-demographic subgroups, contraception use and distribution of induced abortions among Estonians and non-Estonians for the first, second, third, fourth and subsequent abortions were calculated for the periods 1996–2003 and 2004–2011.

**Results:**

Observed trends over the 16-year study period indicated a considerable decline in induced abortions with a reduction in abortion rate of 57.1%, which was mainly attributed to younger cohorts. The percentage of women undergoing repeat abortions fell steadily from 63.8% during 1996–2003 to 58.0% during 2004–2011. The percentage of women undergoing repeat abortions significantly decreased over the 16 years within all selected socio-demographic subgroups except among women with low educational attainment and students. Within each time period, a greater percentage of non-Estonians than Estonians underwent repeat abortions and obtained third and subsequent abortions. Most women did not use any contraceptive method prior to their first or subsequent abortion.

**Conclusion:**

A high percentage of women obtaining repeat abortions reflects a high historical abortion rate. If current trends continue, a rapid decline in repeat abortions may be predicted. To decrease the burden of sexual ill health, routine contraceptive counselling, as standard care in the abortion process, should be seriously addressed with an emphasis on those groups - non-Estonians, women with lower educational attainment, students and women with children - vulnerable with respect to repeat abortion.

## Background

In Estonia, abortion has been legal and accessible during a long period of time. Since 1955, women have been legally allowed to request an abortion up until the 12th week of pregnancy. Termination on medical grounds, including termination in females under 15 years and over 45 years of age, is allowed until the 22nd week of pregnancy. Since 2009, parental consent for minors under 18 years has been required. All abortions are either performed in public hospitals or within the private sector via health insurance schemes for which some of the costs are met by the patient. Despite the high abortion rate during the Soviet Union period [1], abortion was not the subject of any public debate. The issue of abortion is perceived, in Estonian society, as a sexual and reproductive right of women. However, moralistic dilemmas, initiated by conservative political parties and religious organisations, have been periodically raised in Estonia with attempts to undermine abortion rights. It is of paramount importance to have recent and evidence-based knowledge about such sensitive public health issues, in order to try and find practical solutions for avoiding unintended pregnancies. Indeed, statistical data on abortions have been routinely collected by the Estonian Abortion Registry (EAR) since 1996 [2]. Before EAR was established, only aggregate data from medical institutions were collected by Estonian Medical Statistics Bureau.

After regaining independence from the Soviet Union in 1991, Estonia experienced an apparent rapid decline in the number of induced abortions [2]. Nevertheless, recently published data have shown a 2.5 times higher abortion rate in Estonia than the reported total average in the European Union (25.1 vs 10.3, respectively in 2008) [3]. In contrast, the percentage of repeat abortions did not show the same rapid decline and represented a significant proportion of all induced abortions - two out of three abortions in Estonia were obtained by women who had had at least one before [2]. The number of repeat abortions in Estonia is twice as high as that in countries like Sweden, Finland, England and Wales [4-6]. Repeat abortions are largely related to the overall risk of pregnancy. Furthermore, there are several reasons and life situations why some women who have had an abortion are at increased risk of having another [7]. Generally, an abortion is a safe and acceptable solution for terminating an unintended pregnancy. However, repeat abortions may be associated with adverse outcomes in future pregnancies such as preterm births and low birth weights [8,9] and are often preventable: access to sexuality education, effective contraceptive methods and good quality sexual health services are well known measures widely attributed to a decreased prevalence in unintended pregnancies [10,11]. During the last 20 years, Estonia has embarked on a radical transformation of its social and health care system, including education and sexual health services. Sexuality education has been a mandatory part of the Estonian school *curriculum* since 1996 and has had a positive impact on sexual behaviour [12,13]. More than 90% of citizens are covered by social health insurance. Affordable contraceptive methods are available: hormonal contraceptive methods are subsidized by Estonian Health Insurance Fund, which covers 50%; copper IUDs have reimbursement of 100% during one year after delivery. Emergency contraception has been available over-the-counter since 2000. Since effective contraceptive methods became available, the total consumption of hormonal contraceptives has significantly increased [14]. Access to contraceptive care has improved due to implementation of a primary care system with family doctors and midwives becoming responsible for family planning issues. Specific efforts to reach adolescents and young people have been made – in 1991, the first youth counselling centre was initiated to address sexual health issues and contraception, by 2011 the number of the centres increased to 20 [13,15].

Given the high proportion of repeat abortions in Estonia, we can only assume that rapid societal changes may have differentially affected the need for repeat abortions among different subgroups of women and these, in turn, have altered the socio-demographic composition of the population undergoing repeat abortion.

In this study we aimed to describe the overall and age-specific trends of induced abortions in Estonia from 1996 to 2011 with an emphasis on socio-demographic characteristics and the use of contraceptive methods among women who had had repeat abortions. The following questions were asked: a) Has there been a rise in the number of women within any of the socio-demographic subgroups undergoing repeat abortions during the study period? and b) What are the contraceptive patterns of women obtaining their first, second, third, fourth or subsequent abortion? The data about repeat abortions, obtained from the two periods, 1996–2003 and 2004–2011, were compared.

## Methods

### Description of the surveillance system

Calculations utilised data from the EAR [2]. The instrument used for gathering data for the EAR is the Abortion Card, which includes data describing the abortion procedures, as well as each woman's socio-demographic and reproductive background. For every abortion, the Abortion Card is completed by a health care worker by interviewing the patient before the abortion procedure and using case records. All abortions, including spontaneous and induced abortions, e. g. induced abortions for medical reasons, are performed and registered by health care institutions and this is compulsory for all health care institutions providing abortion care, including all private health services. The coding, input, control, correction, saving and processing of data is carried out by the EAR registry on a regular basis. Since 1999, the EAR has not been legally allowed to collect the patient’s personal identification number. Data about live births (based on the Estonian Medical Birth Registry [2]), and the age and ethnic distribution of the study population were obtained from Statistics Estonia [16].

### Induced abortion and repeat abortion data

Only induced abortions were included in the analysis of trends during the period 1996–2011. For the repeat abortion analysis, those abortions performed for medical reasons (3334 [3.0%] during 1996–2003 and 1927 [2.8%] during 2004–2011) were excluded, as were those abortions performed for women with missing information about previous abortions (330 during 1996–2003 and 94 during 2004–2011). The following data are presented in this paper: a) the total number of induced abortions; b) the abortion rate and fertility rate (i.e. the annual number of induced abortions and live births, respectively, among women aged 15–49 years per 1000 women in that age group using the mid-year female population estimates); c) the abortion ratio (i.e. the annual number of induced abortions per 100 live births among women aged 15–49 years); d) the age-specific abortion rates (i.e. the annual number of induced abortions among women in a specific age group per 1000 women using the mid-year female population estimates in the same age group); e) the total abortion rate (i.e. the sum of 5-year age-specific abortion rates for women aged 15–49 years, multiplied by 5, calculated for a period, using the age-specific rates for that period).

### Categorisation of selected socio-demographic characteristics and parity

The following socio-demographic characteristics of women obtaining repeat abortions were analysed: a) distribution by age groups (≤19, 20–24, 25–29, 30–34, 35–39, 40–44, ≥45 years); b) ethnicity (Estonian, non-Estonian); c) educational level (basic/less, secondary, secondary special, university); d) occupation (employed, unemployed, student, other); e) marital status (married, cohabiting, single, divorced/widow); f) parity (0, 1, ≥ 2).

We have presented the total numbers and percentages of women undergoing repeat abortions within the selected socio-demographic subgroups for each study period: 1996–2003 and 2004–2011. Within a selected socio-demographic subgroup the percentage change (with 95% confidence intervals (CI)) of repeat abortions has been calculated as the decrease between two proportions in two observational periods (1996–2003 and 2004–2011) divided by the proportion of this subgroup in the first period.

Distribution of the first, second, third, fourth and subsequent induced abortions among Estonians and non-Estonians is presented during the two time periods.

### Contraception

Contraception use prior to each abortion was assessed by asking: What was the contraceptive method you used before becoming pregnant? The Abortion Card allows the following responses: oral hormonal contraception, intrauterine contraception, condom, other, non-use, no data. The number of times each response was chosen was calculated for the first, second, third, fourth or subsequent abortion during 1996–2003 and 2004–2011.

### Details of ethics approval

Since only statistical data were used, no ethics approval was required for the study.

## Results

### Trends in induced abortion rates, fertility rates and abortion ratios

**Figure 1:**
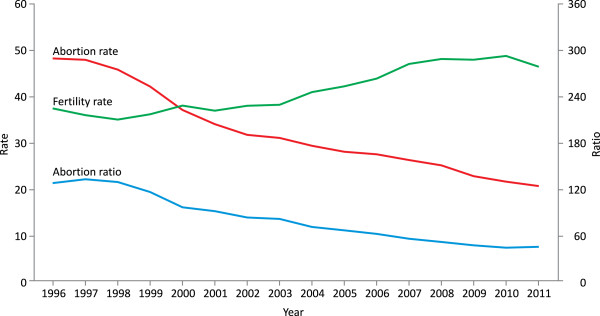
**Fertility rate**^**a**^**, induced abortion rate**^**b **^**and ratio**^**c**^**, Estonia in 1996–2011. **^a^ the annual number of live births among women aged 15–49 per 1000 women aged 15–49 years using the mid-year female population estimates ^b^ the annual number of reported abortions among women aged 15–49 per 1000 women aged 15–49 years using the mid-year female population estimates ^c^ the annual number of reported abortions per 100 live births among women aged 15–49.

Figure 1 presents the trends relating to induced abortion rate, fertility rate and abortion ratio during 1996–2011. In 1996, the annual number of induced abortions was 16 887 equating to an abortion rate of 48.3 and abortion ratio of 128.7. In 2011, respective figures were 6689, 20.7 and 45.0, which represents a reduction in abortion rate of 57.1% and abortion ratio of 65.0%. During the same time period, the total abortion rate dropped from 1.72 to 0.71 (data not shown). Alongside this consistent decline in the number of induced abortion there was a 22.9% increase in fertility rate.

### Age-specific trends

**Figure 2:**
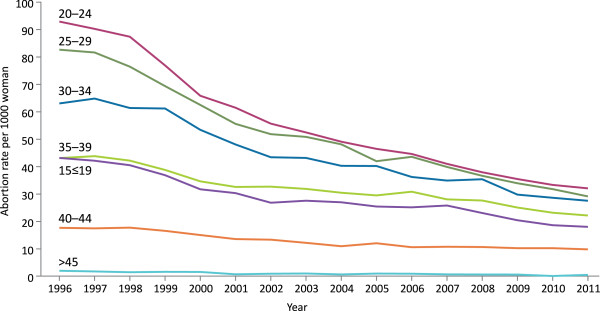
**Age-specific induced abortion rates**^**a**^**, Estonia, in 1996–2011. **^a^ the annual number of induced abortions among women in specific age group per 1000 women using the mid-year female population estimates in the same age group.

The distribution of age-specific induced abortion rates were similar in 1996 and 2011, whereas the abortion rates across all age groups markedly declined over the same period (Figure 2).

Women aged 20–29 years accounted for approximately half of all induced abortion patients (49.4% in 1996 and 47.1% in 2011) having the highest and steepest decline in abortion rates (65.3% among 20–24 year olds and 64.4% among 25–29 year olds) throughout the observed period. Teenagers' (≤19 years) induced abortions accounted for 12.3% of all induced abortions in 1996 and 9.7% in 2011 having a decrease in abortion rate of 56.7%. A smaller decline in abortion rates was observed among older women (55.4% among 30–34 year olds; 47.3% among 35–39 year olds; 41.2% among 40–44 year olds).

For induced abortions on request, repeat abortions were obtained by 63.8% (n = 67 626) of women during 1996–2003 and 58.0% (n = 38 132) during 2004–2011; 26.0% of women during 1996–2003 and 2004–2011 were seeking their second abortion; 17.2% and 15.9% their third; 20.6% and 16.1% their fourth or higher-order abortion.

### Repeat abortions within selected subgroups

**Figure 3:**
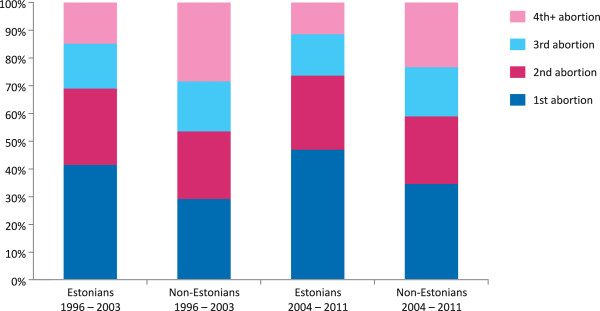
**Percentage of the first, second, third, fourth and subsequent induced abortions**^**a **^**among Estonians and non-Estonians, 1996–2003 and 2004–2011. **^**a**^ only induced abortions on request.

The proportion of women who underwent repeat abortions decreased across all the socio-demographic subgroups during the study period, but most significantly among women under 30 years of age, women with a university degree and nulliparas (Table 1).For women with a basic or less education and students the change was not statistically significant. Although the proportion of women undergoing third and subsequent abortions decreased within both ethnic groups during the study period, it was higher among non-Estonians – the proportion of women undergoing fourth and subsequent abortions was two times higher compared to Estonians (Figure 3).

**Table 1:** Summary of repeat abortions performed within selected socio-demographic and parity subgroups in Estonia, 1996–2003 and 2004–2011

**Characteristic**	**Repeat abortion**				**Percentage change (95% CI**^**d**^**)**
	**1996–2003**		**2004–2011**		
	**N**^**a**^ **= 67 626**		**N**^**a**^ **= 38 132**		
	**n**^**b**^	**%**^**c**^	**n**^**b**^	**%**^**c**^	
**Age (years)**					
≤19	13276	19.7	8500	16.8	-14.6 (-19.4; -9.4)
20–24	26789	48.8	16111	41.5	-15.0 (-16.8; -13.1)
25–29	24402	70.5	14372	63.2	-10.4 (-11.8; -9.1)
30–34	20039	80.3	12503	74.5	-7.2 (-8.3; -6)
35–39	14702	85.9	9883	80.5	-6.3 (-7.4; -5.2)
40–44	6337	88.5	4057	83.9	-5.2 (-6.7; -3.7)
≥45	412	91.3	308	85.7	-6.1 (-11.1; -0.8)
Missing	47		1		-
**Education**					
University	10716	68.2	9107	56.4	-17.3 (-19.1; -15.5)
Secondary	46612	63.6	25443	60.7	-4.5 (-5.6; -3.3)
Secondary special	33766	71.8	16538	67.5	-6.1 (-7.3; -4.9)
Basic/less	14766	43.1	14570	43.7	-1.4 (-1.2; 4.1)
Missing	144		77		-
**Ethinicity**					
Estonian	61222	58.5	40514	53.3	-8.9 (-9.9; -7.9)
Non-Estonians	44471	71.0	25178	65.6	-7.7 (-8.7; -6.7)
Missing	311				-
**Marital status**					
Single	32687	42.1	23164	41.0	-2.4 (-4.3; -0.4)
Married	43085	76.3	17929	71.1	-6.8 (-7.8; -5.8)
Cohabiting	22312	64.8	21067	62.4	-3.7 (-5; -2.3)
Divorced/widow	7787	83.2	3521	76.8	-7.7 (-9.6; -5.7)
Missing	133		54		-
**Occupation**					
Student	12095	21.8	9662	21.1	-3.1 (-8; 2.0)
Employed	61289	72.0	35531	66.4	-7.7 (-8.5; -6.9)
Unemployed	6509	74.3	3829	67.6	-8.9 (-11.3; -6.5)
Other	25718	61.4	16616	59.3	-3.4 (-4.9; -1.8)
Missing	393		97		
**Parity**					
0	25386	30.6	17550	25.8	-15.7 (-18.3; -13)
1	38092	67.4	22801	61.9	-8.1 (-9.2; -7)
2+	42424	80.3	25379	76.8	-4.5 (-5.3; -3.7)
Missing	102		5		

^a^N = total number of women underwent repeat abortion included in each time period;

^b^n = number of women underwent repeat abortion in each subgroup;

^c^% = percentage of women underwent repeat abortion in each subgroup;

^d^CI: confidence interval.

### Use of contraception

Contraception non-use prior to abortion accounted for nearly two thirds of women having a repeat abortion; while the failed use of contraceptive pills and intrauterine contraception was low at the time of first, second, third or fourth or subsequent abortions (Table 2). Condom was the most frequently reported failed contraceptive method prior to all abortions during 2004–2011, and prior to a first or second abortion in 1996–2003. For women undergoing their third, fourth or subsequent abortion during 1996–2003, the most frequently reported contraceptive methods were classified as “other” (rhythm method, withdrawal, spermicides). Within each time period, the proportion of women using “other” contraceptive methods increased as the number of abortions obtained increased, but overall, there was a decrease in these methods over the 16-year period.

**Table 2:** Contraceptive use (%) prior to abortion, Estonia, 1996–2003 and 2004–2011

**Contraceptive method**^**a**^	**1996–2003**				**2004–2011**			
	**N**^**b**^ **= 105 938**				**N**^**b**^ **= 65 735**			
	**1st**	**2nd**	**3rd**	**4th+**	**1st**	**2nd**	**3rd**	**4th +**
**Non-use**	58.5	55.5	54.5	54.7	65.5	63.8	65.0	65.3
**Pill**	4.1	5.6	5.1	4.6	5.1	6.1	6.0	5.2
**IUD**^**c**^	2.4	2.7	2.9	2.2	1.5	1.6	1.6	0.9
**Condom**	18.4	16.3	15.7	14.3	18.1	15.6	14.1	13.4
**Other**	12.0	15.8	17.4	20.3	7.3	10.2	11.0	13.1
**Missing**	5.4	5.2	5.5	5.2	3.0	3.2	2.8	2.8

^a^multiple responses were allowed;

^b^N = total number of women included in each time period;

^c^IUD: intrauterine device.

## Discussion

Our study provided an opportunity to investigate the influence of determinants on abortion trends using complete abortion reporting in a unique situation - legislation and access to abortion have not changed, but political, economic and social changes have been significant during the last two decades in Estonia. Over the 16-year period studied (1996–2011), trends indicated a considerable decline in the number of induced abortions and a slow but consistent decline in the number of repeat abortions undergone in Estonia. The declining abortion rate was mainly attributed to the younger cohorts. The proportion of women undergoing repeat abortions in 2004–2011, compared to 1996–2003, decreased within all socio-demographic subgroups. However, this decrease was not significant among women with a lower level of educational attainment and students. The percentage of non-Estonians undergoing repeat abortions, and of those obtaining third and subsequent abortions was higher than that of Estonians. Most women did not use any contraceptive method prior to their first or subsequent abortions.

### Validity of EAR data

Before these findings are discussed further, we must comment on the completeness and validity of EAR data. For this investigation, we presumed that because it is relatively easy to obtain an abortion in Estonia, virtually no illegally performed abortions occurred. All pregnancies should be documented in case records and diagnosed by ultrasound or hCG-test eliminating procedures called “menstruation regulation” or “miniabortion”, thus ensuring that missing data are minimal within the EAR dataset. When comparing abortion rates between countries, the question of complete reporting will always arise particularly since the accuracy and consistency of reporting impacts directly on the abortion rate reliability [3,10,11]. It has been argued that the decline in induced abortion rates seen in former Soviet countries since 1995 might be overestimated because abortions were increasingly being performed in the private sector so may not have been included in reported statistics [3,10,17]. Therefore, the reliable data collection conducted by the EAR may partly explain why the abortion rate in Estonia, especially compared to other post-Soviet countries, has been reported as the highest in the European Union [3].

### Repeat abortions

In Estonia, the number of repeat abortions, and especially the number of third and higher-order abortions, is high. For instance, in 2011, among all induced abortions, the proportion of women undergoing fourth and subsequent abortions was 17.9% in Estonia, compared to 5.6% in Sweden and <1% in England and Wales [4,5]. A high percentage of women who have undergone repeat abortions reflects a high historical abortion rate. Our analysis revealed that the overall decline in abortion rate observed during the study period was mainly attributed to younger cohorts. Moreover, this observation must be viewed alongside fertility rate: in 1996, the highest fertility rate (i.e. 101 live births per 1000 women of fertile age) was seen among women aged 20–24, but had halved by 2011 to 55.7 and the peak in fertility rate has shifted to women aged 25–35 [2]. The average age of mothers during first births increased from 23.1 in 1996 to 26.3 in 2011 [2]. Currently, the highest percentage of women undergoing repeat abortions are those in their 30’s and 40’s who have either had several abortions in their lifetime, or had a greater number of repeat abortions when younger and showed a lower acceptance of effective contraceptive methods compared to younger women [18]. Thus, we can assume that when older cohorts of women “age out” of their reproductive years, a more rapid decline in the number of repeat abortions is likely to happen in future years. The opposite trend has been observed in many countries during the last 30 years [10,19] and was forecasted by Tietze and Jain: “The proportion of repeat abortions among all legal abortions increases over time as more women in the population have had a first abortion and are, therefore, at risk of having a repeat abortion, until a steady state is reached” [20]. According to the literature, pregnancy unacceptance and contraceptive failure have both been associated with either being single or student [21,22]. This may be one reason why the decrease in the percentage of women undergoing repeat abortions among students and single women was not marked compared to other subgroups, although it should be noted that students and single women represent a small proportion of the overall study population.

### Influence of age, parity and ethnicity

Age is the main predictor for repeat abortion because on the one hand, older women have had more years of exposure to risk of pregnancy and on the other, teenagers who have had one abortion, are at greater risk of having another [6]. It has been argued that women's attitudes and behaviour towards induced abortion are established at a young age and persist during a woman's fertile age [23].

Parity is another key characteristic for repeat abortions because women obtaining repeat abortions are more likely to indicate that they don’t want to have more children [6,19]. In our population 90% of repeat abortions were obtained by parous women*.* However, following the traditional Western European pattern, unwanted pregnancies were terminated before the childbearing commenced, and this can already be seen in abortion statistics during two periods: 23.9% in 1996–2003 and 26.7% in 2004–2011 (p < 0.0001) of all abortions were obtained by nulliparous.

Although less influential than age and parity, ethnic origin has also been associated with repeat abortions [19]. Estonia is ethnically diverse: 34.8% of its population in 1996 and 28.8% in 2011 was composed of non-Estonians, the vast majority of which were Russians [16]. In our dataset, 96.2% of non-Estonians were Russians during 1996–2003 and 97.5% during 2004–2011. Over the last 20 years, the overall abortion rate has been substantially higher and fertility rate lower among non-Estonians compared to Estonians [2]. Non-Estonians were overrepresented among women of fertile age obtaining repeat abortions in 1996 and in 2011 (46.0% and 38.7%, respectively). However, the decrease in repeat abortions among non-Estonians was almost comparable with that among Estonians, while the percentage of non-Estonians obtaining the third or higher order abortions was markedly higher compared to Estonians. One likely explanation, derived from previous studies [18], is that the Russian-speaking women prevented unintended pregnancies by using less reliable contraceptive methods and, in contrast to Estonians, having an abortion increased the risk of them not using contraception in the future.

### Contraception use

Contraceptive patterns before pregnancy termination have been explored in a number of studies [6,19,20] and, according to these data, the variations reflect the differences in overall contraceptive practices across countries. The proportion of women who did not use any contraception prior to their first, second, third and fourth or subsequent abortion, accounted for almost two thirds of the overall population in our study; while this increased over the 16-year period, the use of unreliable contraceptive methods decreased. This might be due to an actual decrease in the use of unreliable contraceptive methods among abortion patients, or these trends may also be explained by an improved knowledge of fertility control. For instance, once the use of the withdrawal or rhythm method fails, women no longer appeared to perceive these approaches as contraceptive methods. The use of condoms was the most frequently reported failed method of contraception prior to abortion and this finding is in accordance with previously published results. Condom use has been shown to have the highest failure rate among contraceptive methods, especially among adolescents, students, single women, and those with no children [19,21,22]. The use of hormonal contraceptive methods prior to abortion was considerably lower than in studies from the US, Finland, France and Denmark [6,19,21,22]. Use of long-acting reversible contraceptive methods, like intrauterine device (IUD) (e.g. copper intrauterine device, levonorgestrel intrauterine system), compared with user-dependent methods, are associated with a lower risk of repeat abortion [6]. This was in agreement with the findings in our study in which IUD users represented the smallest proportion of women having their first or a higher-order abortion. The decrease in the number of IUD users among abortion patients might be the result of an increased use of the reliable levonorgestrel intrauterine system during the study period [14]. However, our findings about pre-abortion contraception are in discordance with the data from other studies from developed countries where the majority of women obtaining their first-time or repeat abortion failed to use a contraceptive method at the time of conception [6,19,21,22,24]. This may be due to variations in study design or may reflect different contraceptive patterns, but is more likely to be influenced by a high overall abortion rate. We can conclude that the majority of abortions in Estonia did not follow contraceptive failures, but occurred because of contraceptive non-use. This confirms that the availability of and access to contraception is not enough to lower the incidence of unintended pregnancies.

A major impact on the abortion rate is the quality of health care services [24,25]. Although there is no robust evidence that contraceptive counselling improves contraceptive adherence and, therefore, reduces the risk of repeat abortion [26,27], there are data to support the fact that having contraception choice, empowers women to make their own decisions and if made at the time of abortion, are important in preventing unintended pregnancies in the future [6,21,26]. An elegant prospective study from Finland showed that immediate initiation of any contraceptive method after abortion, but especially long-acting methods, was linked to a lower risk of repeat abortion [6]. A population-based study in 2004 found that only 24.0% of Estonian women reported receiving pre- or post-abortion contraceptive counselling [28]. In contrast in France, 79.6% of women declared they had received information about contraception before or after abortion [21].

### Study strengths and limitations

Our study’s main limitation was related to the surveillance system not permitting personal identification numbers. This did not allow to link with other data and an analysis of associations between different socio-demographic factors and repeat abortion. Except for age and ethnicity, there were no population-based data about mean annual numbers of other socio-demographic characteristics in Estonia and therefore we couldn’t obtain an exact estimate of how the trends were relative to the population. We are also aware that reliance on the self-reporting of sensitive issues like previous abortions may cause underreporting. However, one study has validated that there is a high degree of completeness in the reporting of recent abortions in Estonia [29] and convinced us that underestimation is minimal. It should also be noted that the Abortion Card only offers five options for contraceptive methods, which includes hormonal contraceptive pills but not transdermal and vaginal hormonal contraceptive methods despite their availability since the early 2000’s. It cannot be guaranteed that misclassification of these hormonal contraceptive methods may have occurred. However, it is our assumption that under the methods named “other” mostly unreliable contraceptive methods such as the rhythm and withdrawal methods and spermicides were classified. Finally, a cross-sectional, population-based study showed that only 6.5% of women who need contraception reported contraception non-use at the time of their last sexual intercourse [18]. This discrepancy with EAR data might be due to a different study sample, different study design, and data collection methods or reflect actual variation in contraceptive practices among women who undergo induced abortion in Estonia. We can only assume that the EAR data largely reflects the actual patterns of contraceptive use at the time of conception.

Despite these limitations, our study provided a unique opportunity to utilise a large, reliable, registry-based dataset to provide a detailed and comprehensive overview of the area of reproduction in Estonia.

## Conclusions

Our study provided evidence that improvements in sexual health services such as sexual education and contraception availability, with an emphasis on providing services for teenagers, have a strong link with abortion rates. Previous abortions are associated with repeat abortions [6,18] and, by default, efforts to reduce unintended pregnancy will reduce the incidence of repeat abortions. A high prevelance of repeat abortions reflects a high abortion rate in the past. If current trends continue, the rapid decline in repeat abortions may be predicted in future years.

There remains a significant unmet need for contraception use, and non-Estonians [18], women with lower educational attainment, students and women with children represent particularly vulnerable groups. During the process of abortion, it is crucial to provide contraceptive counselling as standard care, with an emphasis on initiating contraception immediately after abortion.

A number of questions about repeat abortion remain unanswered and underline the need for further research, particularly into the reasons for contraceptive non-use among women who are at risk of unintended pregnancy. Despite the completeness and reliability of EAR data, there is an urgent need to re-establish data collection that includes personal identification numbers. In addition, qualitative research may be helpful to improve our understanding of women’s attitudes towards childbearing, abortion and contraception in order to find improved practical solutions to avoid repeat abortions.

## Abbreviations

EAR: Estonian Abortion Registry; IUD: Intrauterine device (copper intrauterine device, levonorgestrel intrauterine system).

## Competing interests

The authors declare that they have no competing interests.

## Authors’ contributions

ML: proposed the research idea and design of the study, participated in the data analysis, drafted the manuscript, was responsible for the final preparation of the manuscript. IR participated in the design of the study, performed the statistical analysis. KP: assisted in the research idea, helped to draft the manuscript, was responsible for the final preparation of the manuscript. KA: participated in the data collection and analysis, was responsible for the final preparation of the manuscript. PV: assisted in the design of the study, was responsible for the final preparation of the manuscript. HK: assisted in the research idea and design of the study, was responsible for the final preparation of the manuscript. All authors have commented and approved the final version of the manuscript.

## Pre-publication history

The pre-publication history for this paper can be accessed here:

http://www.biomedcentral.com/1472-6874/14/81/prepub
